# Supplementary material for: Time-restricted eating and supervised exercise for improving hepatic steatosis and cardiometabolic health in adults with obesity: protocol for the TEMPUS randomised controlled trial
Source: BMJ Open. 2024 Jan 24;14(1):e078472. doi: 10.1136/bmjopen-2023-078472 (PMC10824004; doi:10.1136/bmjopen-2023-078472)
Supplement: Supplementary data [file bmjopen-2023-078472supp003.pdf]

HOJA DE INFORMACIÓN PARA EL PARTICIPANTE

DESCRIPCIÓN GENERAL

Nos dirigimos a usted para solicitar su consentimiento para la participación en nuestro estudio con fines de investigación biomédica. El objetivo de este documento es informar sobre el estudio en el cual se le invita a participar. Este estudio ha sido aprobado por el Comité Ético de Investigación Clínica de la Provincia de Granada. El estudio se lleva a cabo con respeto a los principios enunciados en la declaración de Helsinki y a las normas de buena práctica clínica.

Nuestra intención es que usted reciba la información correcta y suficiente para que pueda evaluar y juzgar si quiere o no participar en este estudio. Para ello, lea esta hoja informativa con atención. Estaremos encantados de aclararle todas las dudas que le puedan surgir después de su lectura. Si lo desea, también puede consultar con otras personas (por ejemplo, médicos, familiares o amigos) sobre su participación en este estudio. Si tiene alguna duda diríjase a los investigadores responsables del proyecto.

Debe saber que su participación en este estudio es voluntaria, no tiene contraprestación económica, y que puede decidir no participar o cambiar su decisión y retirar el consentimiento para la utilización de sus datos en cualquier momento, así como solicitar la destrucción de los mismos. Si usted decide revocar su consentimiento, no se realizarán nuevos análisis de sus datos, pero esta revocación no afectará a las investigaciones realizadas hasta el momento.

Si tiene alguna duda diríjase al Investigador Principal del Estudio, el Dr. Jonatan Ruiz Ruiz o el Dr. Manuel Muñoz Torres.

1. DATOS DE LOS INVESTIGADORES RESPONSABLES DEL PROYECTO (ART. 15.2.L.I.B.)

Nombre y apellidos: Jonatan Ruiz Ruiz. **D.N.I:** 46779494R **Centro, teléfono, correo electrónico**: Facultad de Ciencias del Deporte. Universidad de Granada; 958242754; ruizj@ugr.es.

Nombre y apellidos: Manuel Muñoz Torres. **D.N.I:** 00793543C **Centro, teléfono, correo electrónico**: Área de Endocrinología y Nutrición. Hospital Universitario Clínico San Cecilio, Granada; 958246124; mmt@ugr.es

2. PROPÓSITO DEL ESTUDIO (ART. 15.2. L.I.B.)

2.1. Título: Efecto combinado del ejercicio y la restricción horaria de la ingesta sobre la grasa ectópica y la salud metabólica en personas con obesidad: Proyecto TEMPUS

2.2. Propósito del estudio

Este estudio en el que se le invita a participar, tiene como propósito principal estudiar el efecto combinado de un programa de 12 semanas de restricción horaria de la ingesta y ejercicio sobre la grasa ectópica (i.e. grasa hepática, grasa visceral, grasa subcutánea abdominal y grasa intramuscular), la salud cardiovascular y la microbiota intestinal en personas con obesidad. Además, se estudiará la persistencia de los cambios observados en grasa ectópica y factores de riesgo cardiovascular 12 meses después de la finalización de la intervención. El objetivo final del proyecto es proponer una nueva intervención de estilo de vida que sea efectiva para combatir la obesidad a nivel

poblacional, fácil de enseñar a los pacientes durante la práctica clínica diaria, y cómoda y sostenible de seguir en el tiempo. **La restricción horaria de la ingesta nutricional (del inglés, time-restricted eating, en adelante TRE) es una nueva modalidad de ayuno intermitente** que ha despertado un gran interés y expectativas tanto en la comunidad científica, como en la población general. El TRE consiste en comer lo que se quiera, pero en un período concreto del día manteniendo el ayuno durante el resto del día (por ejemplo: comer durante 8 horas y ayunar las 16 horas restantes). Durante el período de ayuno se puede beber agua, infusiones o café solo, es decir, sin azúcar ni edulcorantes (ni artificiales ni naturales). Este régimen dietético se está proponiendo como alternativa al clásico tratamiento de dieta hipocalórica o de restricción calórica para la pérdida de peso. A pesar de que goza de mucha popularidad, todavía hay un gran desconocimiento acerca de su eficacia y también de su viabilidad general.

2.3. Objetivos del estudio

El objetivo del estudio es estudiar el efecto combinado de un programa de 12 semanas que incluye intervención nutricional basada en la reducción del horario de ingesta de alimentos y ejercicio sobre la grasa ectópica y la salud cardiovascular en personas con obesidad.

2.3. Diseño del estudio. ¿En qué consiste el estudio?

Si acepta participar en el estudio, usted será **asignado aleatoriamente** a uno de los siguientes grupos de intervención durante **12 semanas**: (1) TRE, (2) Ejercicio, (3) TRE+Ejercicio, o (4) Grupo Tratamiento Estándar. La aleatorización se realiza utilizando un método estadístico (aleatorización de bloques permutados estratificados) que asigna de forma aleatoria el participante a cada grupo, teniendo en cuenta si el participante es hombre o es mujer para garantizar que los grupos tengan un número similar de hombres y mujeres. Además, el método que se utiliza en la aleatorización garantiza que cuando se asigna una persona a un grupo de los cuatro posibles (grupo tratamiento estándar, Ejercicio, TRE, Ejercicio+TRE) no se pueda adivinar el siguiente grupo que le toca a la siguiente persona. La aleatorización la ha realizado una persona experta en estadística externa al estudio.

Grupos:

1. TRE. Las personas participantes en este grupo realizarán las comidas dentro de una ventana de 8 horas que ellos mismos seleccionarán en base a su actividad laboral y/o social, y ayunarán las 16 horas restantes. Idealmente, la última comida la realizarán antes de las 21:00hrs.

2. Ejercicio. Las personas participantes en este grupo seguirán un programa de ejercicio individualizado 2 días por semana de ejercicio de fuerza de moderada-alta intensidad seguido de un programa de entrenamiento interválico de alta intensidad en el Instituto Mixto Universitario Deporte y Salud. El volumen y la intensidad se irá adaptando semanalmente a la forma física. Además, las personas participantes recibirán una prescripción individualizada de ejercicio aeróbico basado en andar a una intensidad moderada-alta intensidad, cuya progresión será incrementar un 15% los pasos diarios semanalmente.

3. TRE + Ejercicio. Las personas participantes en este grupo seguirán las instrucciones del grupo de TRE, así como del grupo de ejercicio.

4. Grupo Tratamiento Estándar. Las personas participantes en el grupo de tratamiento estándar recibirán recomendaciones generales sobre actividad física

y dieta mediterránea. Estas recomendaciones también se facilitarán al resto de las personas participantes de los otros grupos.

2.4. Intervención del estudio.

El estudio empezará con una fase de 'entrada' (del inglés *run-in period*) al proyecto de dos semanas de duración durante la cual usted mantendrá sus horarios habituales de alimentación y de estilo de vida y registrará los horarios de ingesta y sueño a través de una aplicación móvil y un diario. Durante estas dos semanas, usted también llevará el monitor de glucosa y el monitor de actividad física. Los datos recogidos se analizarán diariamente con el objetivo de (i) confirmar que el horario de ingesta actual es de al menos 11 horas e (ii) individualizar su horario de ingesta al ciclo de sueño/vigilia de en la fase de intervención. Se le recordará la importancia de mantener sus horarios habituales de ingesta y de estilo de vida durante estas 2 semanas de entrada al proyecto.

Una vez finalizado el período de pre-intervención, usted empezará el programa de intervención de 12 semanas en el grupo al que hayan sido asignados. No se permitirá la ingesta de alimentos o bebidas que contengan calorías fuera de la ventana de ingesta de 8 horas de duración en los grupos de TRE y TRE+ejercicio. Se organizarán **3 reuniones** para intercambiar experiencias y tratar y debatir posibles estrategias para hacer frente a los eventos sociales o mejorar hábitos de vida saludable.

2.5. Número de participantes a incluir en el estudio.

Nuestro objetivo es conseguir 184 participantes en total, es decir, 46 participantes por cada grupo, de los cuales en torno al 50% serán hombres y en torno al 50% serán mujeres.

3. VISITAS Y PRUEBAS A REALIZAR.

Si acepta participar, usted tendrá **5 visitas antes, 3 durante la intervención** y otras **3 al finalizar la intervención**, tal y como se describe a continuación. Además, cada dos semanas tendrá las reuniones grupales mencionadas anteriormente.

Día 1. Explicación del estudio, firma del consentimiento informado y evaluaciones de condición física y cuestionarios de nutrición:

Se realizará a cualquier hora del día y sin necesidad de estar en ayunas. Se evaluará la fuerza de prensión manual mediante dinamometría manual, la fuerza del tren inferior mediante el test de levantarse y sentarse de la silla y la prueba de velocidad de la marcha.

Duración aproximada: 90-120 minutos.

Lugar: Instituto Mixto Universitario Deporte y Salud (iMUDS), Parque Tecnológico de la Salud, Granada.

Día 2: Prueba de esfuerzo y colocación de un monitor de glucosa en el brazo y de un monitor de actividad física en la muñeca. Se realizará a cualquier hora del día. En primer lugar, se evaluará la capacidad aeróbica mediante un test caminando en un tapiz rodante y bajo supervisión médica (Dr. Carlos de Teresa Galván o la Dra. Socorro Rebollo Navarrete). Se registrará la actividad cardíaca mediante electrocardiografía para confirmar una respuesta cardiaca adecuada al ejercicio, así como los niveles de lactato, mediante extracción capilar. Posteriormente, le colocaremos un **monitor de glucosa** en el brazo que llevará durante 14 días (totalmente indoloro – necesidad de que en la zona no haya vello), un **monitor de actividad** del tamaño de un reloj que llevará en la muñeca para registrar su actividad física y la duración y calidad del sueño, y una

pulsera de actividad para registrar el número de pasos. También, le entregaremos un **kit** para recoger muestras de **orina** y **heces**, así como las instrucciones pertinentes para hacerlo. Finalmente, le explicaremos cómo registrar sus horarios de comida y sueño a través de una aplicación móvil. Durante estos 14 días usted deberá mantener sus horarios habituales de ingesta y de estilo de vida y registrará cada día la hora a la que realiza la primera y última comida, la hora a la que se va a dormir y se levanta a través de la aplicación móvil.

Duración aproximada: 150 minutos. Este procedimiento se repetirá durante las dos últimas semanas del estudio.

Lugar: Instituto Mixto Universitario Deporte y Salud (iMUDS), Parque Tecnológico de la Salud, Granada.

Día 3. Extracción de sangre, evaluación dietética, psicológica y de la composición corporal: la analítica se realizará en horario de mañana y en ayunas.

Lugar: Hospital Universitario San Cecilio, Granada. Responsable: Manuel Muñoz.

Tras ello podrá dirigirse al iMUDS, para realizar cuestionarios sobre nutrición y psicología. Se medirá el peso, talla, perímetro de cuello, cintura, cadera y pierna, y la presión sanguínea. Analizaremos la composición corporal mediante absorciometría de Energía Dual de Rayos X (DXA) y Bioimpedancia (ambas pruebas totalmente indoloras e inocuas). Además, se le realizará una prueba oral de tolerancia a la glucosa. Tras la ingesta de una disolución de 75 gramos de glucosa anhidra, mediremos tolerancia a la glucosa a través de la extracción de muestras de sangre capilar cada 30 minutos.

Duración aproximada: 180 minutos.

Lugar: Instituto Mixto Universitario Deporte y Salud (iMUDS), Parque Tecnológico de la Salud, Granada. Responsable: Antonio Clavero, Alba Camacho.

Día 4. Resonancia magnética y elastografía (ecografía).

Se realizará a cualquier hora del día. Se tomará una imagen de su abdomen para analizar la cantidad de grasa acumulada en las vísceras y el hígado. La exploración es totalmente indolora e inocua, aunque no se recomienda para personas con algún tipo de **claustrofobia.**

Duración aproximada: 30 minutos.

Lugar: Hospital Universitario San Cecilio, Granada. Responsable: Dr. José Luis Martín Rodríguez y Patricia García.

Día 5. Evaluación dietética, psicológica y de la composición corporal.

Esta visita será llevada a cabo en la mitad de la intervención, tras las primeras 6 semanas. Se realizará en horario de mañana, y en ayunas. Le haremos algunas preguntas acerca de sus hábitos de vida y dietéticos y realizará unos cuestionarios de nutrición y psicología. Se medirá el peso, talla, perímetro de cuello, cintura, cadera y pierna, y la presión sanguínea. Analizaremos la composición corporal mediante absorciometría de Energía Dual de Rayos X (DXA) y Bioimpedancia (ambas pruebas totalmente indoloras e inocuas).

Duración aproximada: 60 minutos.

Lugar: Instituto Mixto Universitario Deporte y Salud (iMUDS), Parque Tecnológico de

la Salud, Granada.

<u>Sesiones de recomendaciones saludables y asignación de grupos.</u>

Se realizarán un total de **3 sesiones.** Se realizará a cualquier hora del día, y sin necesidad de estar en ayunas. Se explicarán las recomendaciones generales sobre actividad física y dieta mediterránea. En la primera sesión, se le indicará a cada participante el **grupo asignado** (de forma **aleatoria**).

<u>Duración aproximada</u>: 60 minutos.

<u>Lugar</u>: Instituto Mixto Universitario Deporte y Salud (iMUDS).

Al finalizar las 12 semanas de intervención, se repetirán los procedimientos previamente explicados en los **días 1-4**. Tras esto, su participación en el estudio habrá finalizado. A los doce meses de la finalización del estudio, donde usted podrá realizar sus horarios de ingesta y hábitos de actividad física como desee, se les contactará para realizar de nuevo algunos procedimientos para ver su evolución. *Las muestras biológicas, así como las pruebas diagnósticas (e.g., resonancia magnética, ecografía, etc.) son realizadas mediante procedimiento expreso para la ejecución del proyecto de investigación.*

4. CRITERIOS DE INCLUSIÓN Y EXCLUSIÓN.

No todas las personas son susceptibles de participar en este estudio. Para ello, recurrimos a la aplicación de **criterios de inclusión y exclusión**. Son una parte muy **importante** de la metodología, y su objetivo es aumentar la probabilidad de que los resultados del estudio sean válidos. Los criterios de inclusión son las características que deben reunir las personas candidatas para considerar su participación en el estudio. Es fundamental reunir todos y cada uno de ellos para ser una persona candidata elegible del estudio. Los criterios de exclusión, por el contrario, definen las características que impiden la participación en el estudio y basta con cumplir alguno de ellos para no poder formar parte del estudio. Además, no debe participar en más de un estudio de investigación sin la aprobación del personal los investigadores involucrados en cada estudio. 3

Tabla 1. Criterios de inclusión y exclusión.

**Criterios de inclusión:**	**Criterios de exclusión:**
• Edad: 25-65 años.<br>• Índice de masa corporal: ≥30,0 y <40,0 kg/m^2.<br>• Un horario medio de ingesta de alimentos auto reportado de al menos 11 horas. El periodo de ingesta comienza con la primera comida y finaliza cuando la última comida esté totalmente terminada.<br>• Peso corporal estable (<5% del peso corporal en la evaluación basal) durante 2 o más meses.	• Antecedentes de un evento cardiovascular grave (Infarto agudo de miocardio, Ictus isquémico o hemorrágico, isquemia arterial periférica), insuficiencia renal, hepatopatía crónica, trastornos del comportamiento alimentario, intervención quirúrgica para el control de peso o VIH / SIDA.<br>• Diabetes mellitus tipo I o II.<br>• Enfermedad endocrinológica activa, errores innatos del metabolismo, miopatías, epilepsia. Pacientes que hayan sido sometidos a técnicas quirúrgicas de cirugía bariátrica o empleadas para el tratamiento de otras patologías (Ejemplo: “Y de Roux”).<br>• Artritis reumatoide, enfermedad de Parkinson, tratamiento activo para el cáncer en el último año,

	u otra afección médica en la que el ayuno está contraindicado.<br>• Uso de medicamentos que pueden afectar los resultados del estudio; por ejemplo, medicamentos para el control de la glucemia (Antidiabéticos orales o insulina, Antihipertensivos, Diuréticos, Corticoides).<br>• Embarazo y lactancia.<br>• Cuidador de una persona dependiente que requiere atención nocturna frecuente/interrupciones del sueño. Trabajadores por turnos variables.<br>• Viajes internacionales prolongados durante el período de estudio.<br>• Abuso activo de tabaco o uso ilícito de drogas o antecedentes de tratamiento por abuso de alcohol (esto es enolismo moderado o severo).<br>• Estar participando en un programa de pérdida de peso, o en un programa de ejercicio estructurado (>30 min en >3 días/semana, o >45min en 2 o más días/semana, a intensidad moderada o alta).<br>• En una dieta especial o prescrita por otros motivos.<br>• Haber realizado ayuno intermitente de forma continuada con anterioridad.<br>• No ser capaz de comprender las instrucciones, objetivos y el protocolo del estudio.<br>• No tener o poder usar un teléfono inteligente con Apple iOS o Android OS.<br>• Cualquier condición que, en opinión del equipo investigador, perjudique la capacidad de participar en el estudio o represente un riesgo personal para el participante.

Contraprestación:

Las personas que participan en el estudio no recibirán ninguna remuneración económica ni material. Sin embargo, se proporcionará toda la información relativa a los parámetros medidos a lo largo de todo el estudio, los cuales ofrecerán información muy valiosa para las personas participantes. A las personas participantes del grupo de tratamiento estándar se les ofrecerá la posibilidad de participar en el programa que haya resultados más eficaces una vez haya finalizado el estudio.

5. INFORMACIÓN SOBRE RIESGOS Y BENEFICIOS.

5.1. Posibles efectos indeseables o secundarios.

En lo relativo a la prueba de esfuerzo:

1. En qué consiste: Consiste en caminar en un tapiz rodante cuya velocidad y/o inclinación se verá incrementada cada minuto, hasta la extenuación.
2. Cómo se realiza: Se realizará por enfermeros, cardiólogo y educadores físico-deportivos con todas las garantías técnicas y salubres.
3. Qué riesgos tiene: Esta prueba no invasiva conlleva un porcentaje muy bajo de complicaciones. No existe riesgo derivado de esta evaluación.

En lo relativo a la extracción sanguínea:

1. En qué consiste: Consiste en la extracción de sangre total mediante punción de una vena periférica, a fin de poder realizar determinaciones de parámetros hematológicos y bioquímicos necesarios para el estudio.
2. Cómo se realiza: Se realizará por enfermeros o médicos con todas las garantías técnicas.
3. Qué riesgos tiene: Cualquier actuación sanitaria tiene riesgos. La mayor parte de las veces estos riesgos no se materializan, en este caso son poco frecuentes y poco importantes.

En lo relativo a la absorciometría de Energía Dual de Rayos X (DXA):

1. En qué consiste: Consiste en la evaluación de la composición corporal mediante Absorciometría de Energía Dual de Rayos X.
2. Cómo se realiza: Durante el escaneo, se tumbará en una camilla que se desliza. Se realizará por personal cualificado con todas las garantías técnicas.
3. Qué riesgos tiene: en este caso no existe ningún riesgo derivado de esta evaluación.

En lo relativo a la prueba oral de tolerancia a la glucosa:

1. En qué consiste: Consiste en ingerir una disolución de glucosa anhidra de 75 gramos para conocer cómo responde su cuerpo a la glucosa.
2. Cómo se realiza: Se realizará por personal cualificado.
3. Qué riesgos tiene: Esta prueba no invasiva no conlleva complicaciones.

En lo relativo a la resonancia magnética y elastografía (ecografía):

1. En qué consiste: Consiste en la realización de una resonancia magnética, la cual usa un gran imán y ondas de radio para analizar la cantidad de grasa acumulada en las vísceras, el hígado, el páncreas y el muslo. Además, se le realizará una ecografía del hígado, totalmente indoloro e inocuo.
2. Cómo se realiza: Durante el escaneo, se tumbará en una camilla que se desliza dentro de un aparato con forma de túnel. Se realizará por personal cualificado con todas las garantías técnicas.
3. Qué riesgos tiene: La resonancia magnética hace mucho ruido, pero el técnico le ofrecerá tapones para los oídos. No existe ningún riesgo derivado de esta evaluación

La intervención de ayuno intermitente y ejercicio no tiene ningún riesgo y no se prevé ningún acontecimiento adverso más allá de los que acompañan en ocasiones a

cualquier régimen nutricional (sensación de hambre, estreñimiento, dolor de cabeza, etc.) o de ejercicio.

5.2. Beneficios esperados.

Algunos estudios de investigación previos indican que la modalidad de ayuno intermitente a implementar en este estudio (TRE) así como la práctica de ejercicio produce una leve/moderada pérdida de peso corporal, así como una mejora de la salud cardiometabólica, concretamente, disminuye la presión sanguínea, el estrés oxidativo y la resistencia a la insulina. Sin embargo, se trata de estudios preliminares que precisan de más estudios confirmatorios.

Al participar en este proyecto, usted tiene derecho a recibir toda la información que se derive de las evaluaciones que le sean realizadas. Si usted lo desea, y nos da su consentimiento, le enviaremos un informe detallado y explicando dichos datos al finalizar el estudio. De igual modo, si alguna de estas evaluaciones presenta resultados que pudieran tener algún impacto sobre su salud, el personal médico e investigador le informará de tal circunstancia y le aconsejará al respecto. Si decide no participar, recibirá todos los cuidados médicos que pudiera necesitar y su relación con el equipo médico e investigador que le atiende no se verá afectada.

6. TRATAMIENTO DE LOS DATOS Y CONFIDENCIALIDAD.

6.1. Tratamiento de datos y confidencialidad.

Los datos recogidos en esta investigación serán anónimos y su uso se regirá por lo recogido en la legislación vigente en relación a la Protección de Datos de Carácter Personal. Los datos personales del participante serán tratados conforme a los términos establecidos en la Ley Orgánica 3/2018 de 5 de Diciembre de Protección de Datos de Carácter Personal, pudiendo ejecutar en cualquier momento los derechos de acceso, rectificación, cancelación u oposición, poniéndose en contacto con el investigador principal según los datos incluidos en el documento de información al participante.

Se solicita su consentimiento para la utilización de sus datos personales (edad, sexo, etnia, etc.) y de salud para investigación. Los datos se recogerán empleando un procedimiento de codificación. Sus datos personales y de salud, serán conocidos por los colaboradores del proyecto con la más estricta confidencialidad durante el desarrollo de este, salvo en la fase analítica del mismo, que será realizada por personal bioinformático ajeno al grupo de investigación, al cual se le entregarán las bases de datos codificadas. Una vez finalizada la fase de análisis, entonces únicamente los IP del proyecto (Drs. Ruiz y Muñoz) podrán correlacionar los resultados con los participantes, siendo anónimo para el resto de los colaboradores del proyecto. La información será procesada durante el análisis de los resultados obtenidos y aparecerá en los informes finales. En ningún caso será posible identificarle personalmente, garantizando la confidencialidad de la información obtenida, en cumplimiento de la legislación vigente.

Los archivos y bases de datos generadas por el estudio se guardarán con estricta confidencialidad. Los archivos físicos se custodiarán bajo llave y con acceso limitado al personal de coordinación del estudio, y la información electrónica se codificará y guardará bajo una carpeta de archivos protegida mediante una contraseña confidencial. En los archivos y bases de datos en los que se disponga de información confidencial o datos de salud no aparecerán el nombre ni los datos personales de las personas participantes, ya que se identificará a las personas participantes mediante un código numérico.

El estudio sigue las directrices de la declaración de Helsinki sobre principios éticos en investigación. Toda su información de carácter personal se tratará con arreglo al Reglamento (UE) 2016/679 del Parlamento Europeo y del Consejo de 27 de abril de 2016 (RGPD) relativo a la protección de las personas físicas en lo que respecta al tratamiento de datos personales y a la libre circulación de estos datos y la Ley Orgánica 3/2018 de 5 de diciembre, de Protección de Personales y garantía de derechos digitales, la Ley 41/2002, de 14 de noviembre, básica reguladora de la autonomía del paciente y de derechos y obligaciones en materia de información y documentación clínica y sus normativas de desarrollo, el Convenio de Oviedo sobre los derechos humanos y biomedicina, así como cualquier norma y/o legislación que le sea de aplicación. Para ello le informamos que: (1) el fin de la recogida y tratamiento de los datos es la gestión del estudio, (2) la legitimación es el consentimiento de los interesados para la realización de las actividades descritas, (3) los datos no se conservarán más tiempo del necesario para la actividad para la cual han sido recabados, excepto que exista una obligación legal, (4) los datos no se comunicarán a otros terceros, excepto obligación legal. Tiene el derecho de retirar el consentimiento, a ejercer el derecho de acceso, rectificación, portabilidad y supresión de sus datos y a la limitación u oposición a su tratamiento, enviando un escrito mediante correo electrónico a los responsables del estudio, asimismo responsables del tratamiento de los datos, Jonatan Ruiz Ruiz (ruizj@ugr.es) o Manuel Muñoz Torres (mmt@ugr.es). Asimismo, tiene derecho a presentar una reclamación ante la Delegada de Protección de datos de la Universidad de Granada, Mª Carmen García Garnica (delegadapd@ugr.es) o ante la Agencia Española de Protección de Datos en caso de incumplimiento por parte de la entidad. Los resultados obtenidos de este estudio pueden ser publicados en revistas científicas de manera anonimizada y la identidad de los/las participantes nunca y bajo ningún concepto serán facilitados.

6.2. Uso potencial de los resultados de la investigación en el futuro, incluido el comercial:

Las muestras biológicas serán tratadas de manera codificada durante su análisis, quedando la base de datos que correlaciona el código con la identificación de la persona participante bajo el control y conocimiento de los IP del proyecto.

Usted podrá decidir el destino del excedente de la muestra biológica que pudiera quedar marcando para ello la opción correspondiente en el cuadro de consentimiento, de entre las siguientes:

- ☐ **Destrucción**.
- ☐ **Anonimización** de la muestra para usos posteriores con fines de investigación biomédica, en cuyo caso no podrá relacionarse con usted, sin cesión al Biobanco del Sistema Sanitario Público de Andalucía. El excedente de la muestra también puede añadirse a otra línea de investigación relacionada con la investigación inicial, incluso la de un tercero mediante cesión gratuita.
- ☐ **Cesión gratuita al Biobanco del SSPA (Código**: S2300146). Las muestras se procesarán y se custodiarán hasta ser solicitadas por el investigador principal en las instalaciones del Nodo Granada del BBSSPA (Biobanco ibs. Granada). La muestra será **codificada**, sometiéndose a un procedimiento para evitar la asociación entre la muestra y usted, sustituyéndose su información de identificación, por un código. La información completa sólo estará a disposición de dicho biobanco, ni siquiera el personal sanitario que lo atiende tendrá acceso a ella. Podrá solicitar su eliminación en cualquier momento. En caso de que se

produzca el cierre o la revocación de la autorización para la constitución y funcionamiento del Biobanco del SSPA, la información sobre el destino de las muestras estará a su disposición en el Registro Nacional de Biobancos para Investigación Biomédica, con el fin de que pueda manifestar su conformidad o disconformidad con el destino previsto para la muestra donada. La persona responsable de la colección privada (Drs. Ruiz y Muñoz, IPs del proyecto) pondrá a disposición del donante toda la información sobre los distintos proyectos de investigación en los que se utilice la muestra.

Usted tiene que decidir libremente si participa o no en el proyecto de investigación. Si decide participar se le proporcionará esta hoja de información y deberá firmar el consentimiento para su participación en el mismo. Así mismo se le entregará una copia de este documento y el investigador mantendrá una copia con el documento original.

El equipo de investigación le agradece su colaboración y participación en el estudio.

CONSENTIMIENTO INFORMADO DEL PARTICIPANTE

(Los artículos se refieren a la Ley de Investigación Biomédica, BOE 4 de julio de 2007)

D./Dª……………………………………………………………………………………

con D.N.I. ………………………………………… y fecha de nacimiento……………………

Declaro que:
He leído (o me han leído) la hoja de información del proyecto titulado **Efecto combinado del ejercicio y la restricción horaria de la ingesta sobre la grasa ectópica y la salud metabólica en personas con obesidad: Proyecto TEMPUS** en España, que me ha entregado el investigador responsable del proyecto: Dr. D. Jonatan Ruiz Ruiz con DNI 46779494-R y Manuel Muñoz Torres con DNI 00793543C.

2. He comprendido la investigación que se va a realizar con mi participación y he tenido la oportunidad de resolver cualquier duda al respecto.

3. Así mismo, se me ha informado de que:
- Se hace constar que el participante manifiesta expresamente decir la verdad en sus respuestas para garantizar los datos reales sobre su estado físico o salud o los que se le solicitan (art. 23.1. L.I.B).
- Tengo derecho a no otorgar mi consentimiento a participar y a revocarlo en cualquier momento del estudio (art. 4.3. L.I.B.)
- La falta de consentimiento a iniciar el estudio o su revocación una vez iniciado no me supondrá perjuicio alguno (en cualquier otro derecho) o discriminación (art. 4.4 y 6. L.I.B.).
- Seré informado, si así lo deseo, de los datos que se obtengan durante la investigación (art. 4.5 y 27.2. L.I.B.) y de la forma de obtener dicha información (art. 15.2. L.I.B.).

4. Acepto participar voluntariamente en el proyecto antes mencionado, con lo que doy autorización a que se me realice lo siguiente:
- Evaluación de antropometría, presión arterial y composición corporal mediante Densitometría Dual de Rayos X y Bioimpedancia.
- Evaluación de la grasa visceral y del hígado mediante resonancia magnética y ecografía.
- Extracción de sangre en vena (volumen total <60 mL de sangre) y capilar (prueba oral de tolerancia a la glucosa y determinación de lactato).
- Evaluación dietética y psicológica mediante cuestionarios.
- Evaluación de la actividad física y de la glucemia con monitores portátiles.
- Recogida de muestras de orina y heces.
- Evaluación de la capacidad aeróbica y de la fuerza muscular.

5. Doy mi autorización para que me contacte en futuros estudios de investigación.

6. He comprendido que mi participación no tiene ninguna contraprestación económica.

7.Autorizo la utilización de imágenes con fines docentes y científicos con absoluto respeto a mi intimidad.

Firma del participante Fecha y lugar

Fdo: ……………………………………………………..

Firma del investigador
Fdo-. Jonatan Ruiz Ruiz – Manuel Muñoz Torres.

REVOCACIÓN DEL CONSENTIMIENTO INFORMADO

D./Dª………………………………………………………………………………………

con D.N.I. ………………………………………………………, declara que:

1. He leído la Hoja de Información y Revocación del Consentimiento Informado que me ha sido entregada.

2. He hablado y aclarado las posibles dudas sobre mi revocación con el Dr./Dra.

………………………………………………………………………………………………

3. Revoco el consentimiento anteriormente prestado por lo que queda sin efecto a partir de este momento

4. Mi revocación es (índique cual):

- ☐ Total.
- ☐ Parcial.

En …………………….. a …. de …………… de 20….

Fdo: ………………………………………………………..

<u>Relativo a los familiares/tutores/representantes legales:</u>

D./Dª………………………………………………………………………………

……, con D.N.I.................................., no tiene capacidad de decidir en este momento.

Por lo que D./Dª. …………………………………………………………………….,

con D.N.I .... y en calidad de.. revoco el consentimiento anteriormente prestado por lo que queda sin efecto a partir de este momento.

En ……………... a ……. de …………… de 20…….

Fdo: ………………………………………………………..

Junta de Andalucía
Consejería de Salud y Familias
BIOBANCO DEL SISTEMA SANITARIO PÚBLICO DE ANDALUCÍA

Consentimiento informado para la donación de muestras biológicas y/o autorización de acceso a datos para un proyecto específico gestionado por el Biobanco del Sistema Sanitario Público de Andalucía y uso posterior del sobrante de muestras en otros proyectos.

DATOS DEL/DE LA DONANTE Y DE SU REPRESENTANTE (éste último sólo en caso de incapacidad del/de la donante):

Apellidos y nombre del/de la Donante:

……

DNI / NIE: …………………………………

Apellidos y nombre del/de la representante legal (cuando proceda)**:**

………………………………………………………………..…………………………………………………………………........

DNI / NIE: ………………………………...

PROFESIONALES QUE INTERVIENEN EN EL PROCESO DE INFORMACIÓN Y/O CONSENTIMIENTO:

Los siguientes profesionales declaran que se ha explicado la información relativa a la donación de muestras biológicas:

Apellidos y nombre

………………………………………………………………………

DNI / NIE: ………………………………...

CONSENTIMIENTO:

Yo, D./Dña. ……………………………………………………………………………………………….declaro que **he leído y comprendido el documento informativo**, que acompaña a este consentimiento y del que se me ha entregado un ejemplar.

He **recibido suficiente información** sobre el estudio Efecto combinado del ejercicio y la restricción horaria de la ingesta sobre la grasa ectópica y la salud metabólica en personas con obesidad (*Título del proyecto*) y sobre el Biobanco del Sistema Sanitario Público de Andalucía que podrá entregar las muestras y datos para otros proyectos relacionados con la misma línea o área de investigación garantizando siempre el cumplimiento de la legislación vigente.

(Indicar Línea/Área Investigación según capítulos CIE-10):

I00-I99

Usted autoriza a que las muestras y datos sobrantes puedan ser cedidas también a proyectos de las siguientes áreas o líneas de investigación:

- ☐ Ciertas enfermedades infecciosas y parasitarias (A00-B99)
- ☐ Neoplasias (C00-D49)
- ☐ Enfermedades de la sangre y órganos hematopoyéticos y ciertos trastornos que afectan al mecanismo inmunológico (D50-D89)
- ☐ Enfermedades endocrinas, nutricionales y metabólicas (E00-E89)
- ☐ Trastornos mentales, del comportamiento y del desarrollo neurológico (F01-F99)

Ejemplar para Biobanco

Camacho-Cardenosa A, *et al. BMJ Open* 2024; 14:e078472. doi: 10.1136/bmjopen-2023-078472

Junta de Andalucía
Consejería de Salud y Familias
BIOBANCO DEL SISTEMA SANITARIO PÚBLICO DE ANDALUCÍA

- ☐ Enfermedades del sistema nervioso (G00-G99)
- ☐ Enfermedades del ojo y sus anexos (H00-H59)
- ☐ Enfermedades del oído y de la apófisis mastoides (H60-H95)
- ☐ Enfermedades del aparato circulatorio (I00-I99)
- ☐ Enfermedades del aparato respiratorio (J00-J99)
- ☐ Enfermedades del aparato digestivo (K00-K95)
- ☐ Enfermedades de la piel y del tejido subcutáneo (L00-L99)
- ☐ Enfermedades del aparato musculoesquelético y del tejido conectivo (M00-M99)
- ☐ Enfermedades del aparato genitourinario (N00-N99)
- ☐ Embarazo, parto y puerperio (O00-O9A)
- ☐ Ciertas afecciones originadas en el periodo perinatal (P00-P96)
- ☐ Malformaciones congénitas, deformidades y anomalías cromosómicas (Q00-Q99)
- ☐ Lesiones traumáticas, envenenamientos y otras consecuencias de causas externas (S00-T88)

Así mismo, consiente que las muestras puedan ser utilizadas para otros fines:

- ☐ Docencia
- ☐ Control de calidad

He podido hacer preguntas sobre la información recibida y hablar con el profesional indicado, quien me ha resuelto todas las dudas que le he planteado.

Deseo que dichas muestras y los datos clínicos asociados se incorporen en el Biobanco de forma:

☐ **Codificada** (serán identificadas con un código que protege mi identidad, siendo posible volver a ligarlas conmigo) o

☐ **Anonimizada** (no se podrán asociar las muestras conmigo, por haberse eliminado de forma irreversible la vinculación entre las mismas y mi identidad).

Autorizo que se pueda **contactar conmigo posteriormente** **(siempre que no se trate de muestras anonimizadas)**:

☐ SI

☐ NO

En caso afirmativo, por favor, indique los medios para hacerlo:

Teléfono: *(indicar número)*…………………………………………………………………….

Correo electrónico: *(indicar dirección)*……………………………………………………….

Otros: (identificar)…………………………………………………………………………………

Ejemplar para Biobanco

Junta de Andalucía
Consejería de Salud y Familias
BIOBANCO DEL SISTEMA SANITARIO PÚBLICO DE ANDALUCÍA

Autorizo a **recibir información** sobre datos genéticos y/o datos relevantes para mi salud (si solicita que las muestras sean anonimizadas, no podrá recibir esta información)
Marque lo que proceda:

☐ SI
☐ NO

Comprendo que puedo retirarme del estudio o retirar las muestras del Biobanco del SSPA:

- Cuando quiera
- Sin tener que dar explicaciones
- Sin que repercuta en mis cuidados médicos.

Presto libremente mi conformidad a participar en el proyecto informado y al tratamiento de mis muestras y datos en los términos informados.

En , a de de

EL/LA DONANTE	EL/LA REPRESENTANTE LEGAL (sólo en caso de incapacidad del/de la donante)
Fdo.:	Fdo.:

PROFESIONAL QUE INFORMA
Fdo.:

Ejemplar para Biobanco
