# Supplementary material for: Time-restricted eating and supervised exercise for improving hepatic steatosis and cardiometabolic health in adults with obesity: protocol for the TEMPUS randomised controlled trial
Source: BMJ Open. 2024 Jan 24;14(1):e078472. doi: 10.1136/bmjopen-2023-078472 (PMC10824004; doi:10.1136/bmjopen-2023-078472)
Supplement: Supplementary data [file bmjopen-2023-078472supp001.pdf]

Movement pattern	Exercise	Graphical representation	Level 1	Level 2	Level 3	Level 4
**Horizontal push**	Push-up	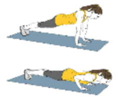	Wall push-up 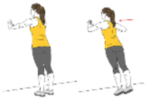	Incline push-up 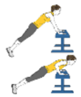	Knee push-up 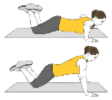	Push-up 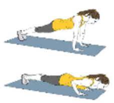
**Vertical pull**	Lat pulldown with resistance elastic bands	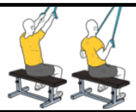	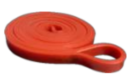	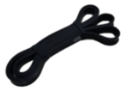	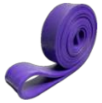	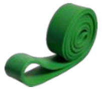
**Hip-dominant**	Glute bridge	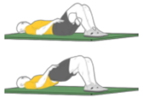	Basic 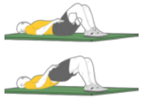	With step 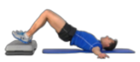	Single leg 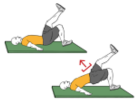	With fitball 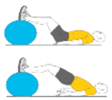
**Knee-dominant**	Squat	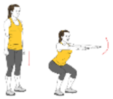	Chair squat 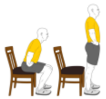	Half squat 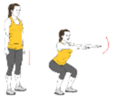	Full squat 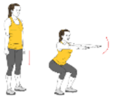	
**Vertical push**	Seated shoulder press with resistance elastic bands	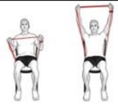	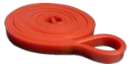	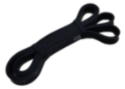	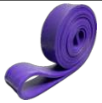	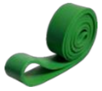
**Horizontal pull**	Seated row with resistance elastic bands	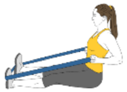	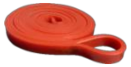	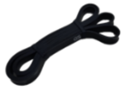	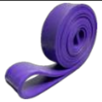	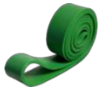
**Knee-dominant**	Lunge	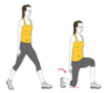	Static Lunge 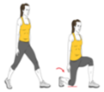	Alternating Lunge 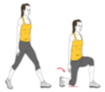	Walking Lunge 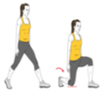	
**Hip-dominant**	Deadlift with resistance elastic bands	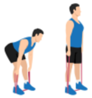	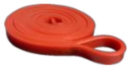	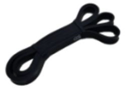	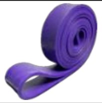	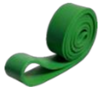

Supplemental Figure 1. Graphical representation of the 4 intensity levels for each exercise. The resistance elastic bands provide varying loads based on their degree of elongation: red band, ranging from 6.8 to 15.9 kg; black band, from 11.3 to 29.5 kg; purple band, from 15.9 to 38.6 kg; green band, from 24.9 to 56.7 kg.
