# Supplementary material for: Time-restricted eating and supervised exercise for improving hepatic steatosis and cardiometabolic health in adults with obesity: protocol for the TEMPUS randomised controlled trial
Source: BMJ Open. 2024 Jan 24;14(1):e078472. doi: 10.1136/bmjopen-2023-078472 (PMC10824004; doi:10.1136/bmjopen-2023-078472)
Supplement: Supplementary data [file bmjopen-2023-078472supp002.pdf]

Example of a exercise training session		
**Resistance training**	**Warm-up**	5 min
	Circuit Lap	8 min
	Rest	2 min
	Circuit Lap	8 min
	Rest	2 min
	Circuit Lap	8 min
	Rest	2 min
	Circuit Lap	8 min
**Resting period between resistance and HIIT**		3-5 min
**HIIT**	**Warm-up**	2 min
	Vigorous-intensity	4 min
	Moderate-intensity	4 min
	Vigorous-intensity	4 min
	Moderate-intensity	4 min
	Vigorous-intensity	4 min
	Moderate-intensity	4 min
	Vigorous-intensity	4 min
	Moderate-intensity	4 min
**Cool down**		5 min

Supplemental Figure 2. Example of a resistance training session performed in the week 11 and 12 of the TEMPUS project. HIIT: high-intensity interval training.

Camacho-Cardenosa A, *et al. BMJ Open* 2024; 14:e078472. doi: 10.1136/bmjopen-2023-078472
